# Obesity and its health implications: The role of metabolic and bariatric surgery in improving physical, mental, and psychosocial well-being

**DOI:** 10.1038/s41366-026-02055-w

**Published:** 2026-03-27

**Authors:** Giorjines Boppre, Andrea Bezerra, Andrés Baena-Raya, Alba Hernández-Martínez, Alberto Soriano-Maldonado, Rodrigo Zacca, Hélder Fonseca

**Affiliations:** 1 Institute of Molecular Sports and Rehabilitation Medicine, Paracelsus Medical Private University, Salzburg, Austria; 2 Centro Regional de Estudios Avanzados en Estilos de Vida Activos y Saludables (CREA-EVAS), Universidad Adventista de Chile, Chillán, Chile; 3 Research Center in Physical Activity, Health and Leisure (CIAFEL), Faculty of Sport, University of Porto (FADEUP), Porto, Portugal; 4 Laboratory for Integrative and Translational Research in Population Health (ITR), Porto, Portugal; 5 SPORT Research Group (CTS-1024), CIBIS (Centro de Investigación para el Bienestar y la Inclusión Social) Research Center, University of Almería, Almería, Spain; 6 Department of Medicine, Division of Endocrinology & Einthoven Laboratory for Experimental Vascular Medicine, Leiden University Medical Center, Leiden, Netherlands; 7 Department of Preventive Medicine and Public Health, School of Medicine, Universidad Autónoma de Madrid, Spain; CIBER of Epidemiology and Public Health (CIBERESP), Madrid, Spain, Universidad Autónoma de Madrid, Madrid, Spain; 8 Department of Education, Faculty of Education Sciences, University of Almería, Almería, Spain

**Keywords:** Obesity, Weight management, Obesity

## Abstract

Obesity is a chronic, heterogeneous disease with profound physical, mental, and psychosocial consequences,requiring eff ective therapeutic strategies beyond lifestyle and pharmacological approaches. Metabolic and bariatricsurgery (MBS) has emerged as the most eff ective treatment for severe obesity, inducing substantial and sustainedweight loss alongside improvements in cardiometabolic health, quality of life, and psychosocial well-being. However,long-term outcomes vary, with some patients experiencing weight regain and persistent psychosocial challenges.This Perspective highlights the additive role of structured exercise and emphasizes the need for continuouspsychological and multidisciplinary support, positioning MBS within integrated, long-term care frameworks tooptimize patient outcomes.

Obesity is a global health crisis affecting more than one billion individuals across different age groups and socioeconomic contexts. Projections suggest that prevalence will continue to rise, creating a profound challenge for health systems [1]. More recently, updated definitions and diagnostic criteria for clinical obesity have been proposed, highlighting the importance of excess adiposity in combination with obesity-related complications and functional impairment, rather than reliance on body mass index alone. This framework conceptualizes obesity as a chronic condition with heterogeneous clinical expression [2]. Epidemiological evidence demonstrates that grades II and III obesity are particularly linked to cardiovascular disease, type 2 diabetes, several forms of cancer, and reduced life expectancy [3]. These findings emphasize the heavy toll of obesity not only on physical health but also on social participation, productivity, and quality of life. Addressing obesity, therefore, requires effective therapeutic strategies alongside preventive action at the population level.

Lifestyle interventions remain the cornerstone of management but have proven insufficient in sustaining long-term weight reduction for most individuals with severe obesity. Pharmacotherapy is advancing but remains costly and not universally accessible. Metabolic and Bariatric Surgery (MBS) has thus emerged as the most effective treatment option for patients with higher grades of obesity or for those with moderate obesity complicated by comorbidities. Over the past two decades, Roux-en-Y gastric bypass (RYGB) and sleeve gastrectomy have become the dominant procedures worldwide [4]. International consensus guidelines from leading surgical societies recommend these operations for patients with a body mass index ≥35 kg/m² or ≥30 kg/m² with comorbidities, with lower thresholds for some populations [4]. These recommendations reflect extensive evidence supporting surgery as a safe, effective, and durable therapy.

The MBS benefits are well established, and patients frequently achieve substantial and sustained weight loss, which is accompanied by remission of type 2 diabetes, improvement in hypertension, and normalization of lipid profiles [4]. Obstructive sleep apnea shows particularly high remission rates following RYGB. These improvements are driven by a combination of caloric restriction, altered nutrient absorption, hormonal modulation, and changes in bile acid metabolism. Collectively, these adaptations reshape energy balance and glucose regulation, demonstrating the capacity of surgery to modify disease mechanisms at a fundamental level.

Equally important are the psychosocial benefits that accompany surgical weight loss. Patients often report greater self-esteem, improved body image, and enhanced quality of life. Sexual health outcomes improve as well, with increased desire, satisfaction, and restoration of fertility in women [5, 6]. These changes reflect both biological factors, such as normalization of sex hormones, and psychological relief from stigma and social exclusion. Patients often describe improved social integration and stronger interpersonal relationships following MBS, reflecting the broader psychosocial transformation that accompanies physical change [7]. The mental health dimension of MBS is more complex. Evidence from systematic reviews indicates that many patients experience reductions in depressive symptoms and anxiety after surgery [8], particularly those with a prior history of mental illness. Enhanced life satisfaction and self-efficacy are frequently reported, often linked to restored functional capacity and the ability to engage in daily activities. Nevertheless, mental health outcomes are not universally positive. Some individuals continue to struggle with body image dissatisfaction, even after major weight loss, and there is evidence of elevated risk of self-harm or suicide compared with BMI-matched controls. These findings underscore the necessity of continuous psychological support and highlight that surgery alone cannot resolve all aspects of obesity as a biopsychosocial condition.

Exercise is a vital component in maximizing the long-term benefits of MBS. As patients lose weight and mobility improves, they gain greater capacity to participate in structured physical activity. Randomized controlled trials demonstrate that exercise following surgery provides additional advantages beyond the surgical effect alone. These include further reductions in fat mass, preservation of lean tissue, improved muscle strength, and better cardiometabolic profiles. Exercise training enhances cardiorespiratory fitness, lowers blood pressure, and improves lipid metabolism. Beyond these physiological effects, participation in exercise supports greater independence and contributes to psychological well-being, reinforcing the positive cycle of recovery. Recent trials have confirmed these benefits in rigorous settings. In one randomized controlled trial, Boppre and colleagues demonstrated that supervised exercise training improved body composition after MBS, preserving lean mass while enhancing fat loss [9]. In a subsequent trial, the same group showed that a multicomponent program targeting aerobic capacity, resistance training, and functional exercise significantly increased muscle strength [10]. These findings confirm the additive value of structured exercise and support the integration of exercise professionals into interdisciplinary care teams. To illustrate this integrated approach, Fig. 1 provides a conceptual overview of the multidimensional framework through which MBS, supported by structured exercise and counseling, contributes to physical, mental, and psychosocial outcomes.

**Fig. 1:**
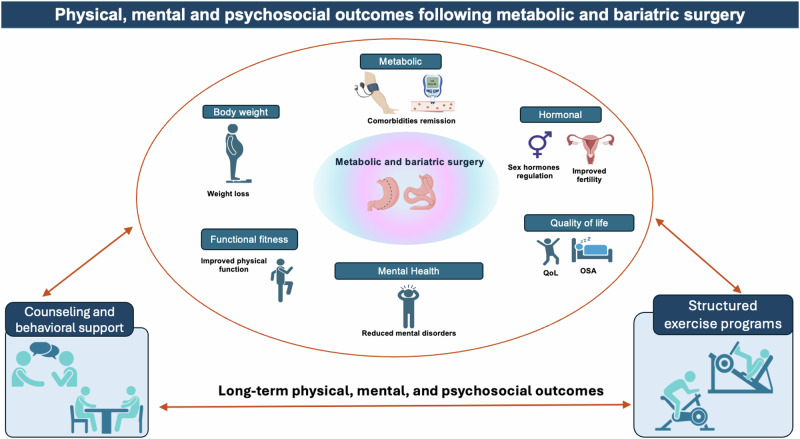
Conceptual framework illustrating the multidimensional effects of MBS on physical, mental, and psychosocial well-being. The figure highlights the central role of MBS, with structured exercise programs acting as a key amplifier of metabolic health, functional capacity, and body composition, supported by counseling and behavioral follow-up to sustain long-term outcomes. QoL Quality of Life, OSA obstructive sleep apnea.

Despite these advances, challenges remain; a proportion of patients experience weight regain in the years following surgery, and outcomes vary widely across populations [7]. Factors such as adherence to dietary guidance, engagement in exercise, and continuity of psychological support strongly influence long-term success [8]. Some patients face persistent body image concerns or struggle with excess skin, both of which can undermine perceived progress. These issues highlight the importance of ongoing multidisciplinary follow-up that addresses not only physical parameters but also psychosocial adaptation.

Looking ahead, the integration of MBS into broader models of chronic disease management offers an opportunity to reshape obesity care. Future research should focus on identifying predictors of surgical success, including genetic, metabolic, and behavioral markers, to enable more personalized treatment strategies [4]. Long-term studies examining not only weight trajectories but also quality of life, mental health, and functional capacity are needed [7, 8]. Equally, greater emphasis should be placed on widely applicable exercise interventions and digital platforms that provide continued support beyond the hospital setting [9, 10]. As the prevalence of obesity continues to rise, the value of MBS will increasingly depend on its integration into holistic care frameworks that combine surgical efficacy with sustainable lifestyle modification and psychosocial support.

Therefore, MBS represents far more than a procedure for weight reduction and remission. It is a transformative therapy that improves metabolic disease, restores psychological well-being, and fosters social reintegration. The addition of structured exercise programs amplifies these effects [9, 10], while ongoing counseling and support help sustain them over time [7, 8]. As evidence accumulates, the imperative is clear: surgery should be considered not in isolation but as a central component of a interdisciplinary approach that addresses the biological, mental, and social dimensions of obesity. This integrated perspective holds the greatest promise for improving the health and well-being of individuals living with severe obesity.
